# Engineered nanocarriers for targeted lung cancer therapy: mechanistic innovations and recent clinical progress

**DOI:** 10.37349/etat.2025.1002339

**Published:** 2025-10-12

**Authors:** Heayyean Lee, Khadijah Sajid, Jeehoo Lee

**Affiliations:** IRCCS Istituto Romagnolo per lo Studio dei Tumori (IRST) “Dino Amadori”, Italy; ^1^Department of Research and Development, Plamica Labs, Boston, MA 02163, USA; ^2^Department of Internal Medicine, St. Luke’s University Health Network, Bethlehem, PA 18015, USA

**Keywords:** lung cancer, nanomedicine, tumor microenvironment, inhalable nanomedicine, stimuli-responsive delivery, nano-immunotherapy, translational nanotechnology

## Abstract

Lung cancer remains the leading cause of cancer mortality worldwide, with progress limited by tumor heterogeneity, drug resistance, and conventional therapy limitations. Nanotechnology-enabled drug delivery offers a transformative approach, enabling the precise engineering of nanocarriers for selective targeting, controlled release, and reduced toxicity. Recent innovations include inhalable systems that achieve localized pulmonary deposition, stimuli-responsive nanocarriers that release drugs in response to tumor microenvironment cues, and nano-immunotherapies that synergize with immune checkpoint blockade. Exosome-based vesicles further offer biomimetic advantages of low immunogenicity and natural tissue tropism. In parallel, theranostic platforms integrate treatment with imaging to enable real-time monitoring of drug delivery and tumor response. This review synthesizes mechanistic advances and translational developments in lung cancer nanomedicine, with emphasis on strategies that overcome biological barriers such as hypoxia, extracellular matrix density, and efflux pump activity. Clinical progress between 2020 and 2025 highlights next-generation antibody—drug conjugates, nanoparticle vaccines, and gene-loaded systems, several of which have reached regulatory approval or advanced trial stages. Together, these advances highlight the potential of nanocarriers to transform lung cancer therapy into more precise, personalized, and less toxic interventions.

## Introduction

Lung cancer remains the leading cause of cancer-related mortality worldwide, with approximately 2.5 million new cases and 1.8 million deaths in 2022 [1]. Although targeted therapies and immune checkpoint inhibitors have improved outcomes, efficacy is frequently limited by intrinsic or acquired resistance and by suboptimal delivery to tumor sites [2]. Standard treatments, including surgery, chemotherapy, and radiotherapy, provide limited survival benefits in metastatic lung cancer and are associated with significant systemic toxicity [3]. The complex tumor microenvironment (TME) imposes physical and biological barriers that hinder drug penetration and therapeutic efficacy [4]. Clinically, current treatment strategies remain inadequate for many patients, as durable responses are rare and treatment-related toxicities are severe. This situation highlights an urgent need for therapeutic platforms that can enhance efficacy while minimizing systemic side effects, thereby improving patient quality of life.

Nanotechnology designs materials at the 1–100 nm scale, yielding nanoparticles (NPs) with physicochemical properties that are distinct from those of their bulk counterparts. NPs can be fabricated from lipids, polymers, metals, or other materials, and their small size confers a high surface area and tunable surface chemistry [5]. Such nanoscale systems differ from their bulk counterparts in exhibiting novel optical, electrical, and biological behaviors that make them highly attractive for biomedical innovation [6]. These attributes enable improved drug solubility, stability, and controlled biodistribution, making NPs powerful tools in medical applications [7]. Nanomedicine offers a promising solution by enabling precise control over drug pharmacokinetics, biodistribution, and tumor targeting. Engineered nanocarriers are designed with molecular-level precision and functional surface modifications to enhance tumor accumulation, thereby improving selective uptake by cancer cells [8]. Functionalizing the NP surface with targeting ligands enables active binding to overexpressed receptors on cancer cells, increasing on-target delivery and reducing off-target toxicity. These nanocarriers can also be tailored to release their therapeutic payload in response to tumor-specific stimuli, such as acidic pH, enzyme activity, or externally applied triggers, thereby ensuring that drug action is concentrated at the tumor site while minimizing harm to surrounding healthy tissue [9]. Moreover, co-delivery of multiple therapeutic agents within a single NP can address tumor heterogeneity and multidrug resistance (MDR) [10]. Many of these nanocarriers are adaptable for inhalable pulmonary delivery, achieving high local drug concentrations in the lungs with minimal systemic exposure and an improved therapeutic index. Notably, several nano-formulations have already progressed into clinical trials, with a few achieving regulatory approval, underscoring their translational potential [11].

Given these developments, this review aims to provide an integrative and up-to-date synthesis of recent progress in lung cancer nanomedicine. We focus on a diverse range of nanocarrier strategies, including inhalable systems, TME-responsive “smart” NPs, nano-immunotherapeutic approaches, and theranostic nanotechnologies, that collectively tackle longstanding challenges such as drug resistance and off-target toxicity. Our scope goes beyond prior reviews by uniting these facets into a comprehensive translational perspective. In particular, we incorporate the latest clinical trial data and regulatory developments up to 2025, offering a timely overview of how nano-enabled therapies are advancing from the bench to the bedside. Through this integrative approach, we provide a distinct bench-to-bedside outlook that links mechanistic innovation with clinical application.

## Biological barriers and nanocarrier design strategies in lung cancer

Drug delivery in lung cancer is often limited by multiple biological barriers, including significant tumor heterogeneity, MDR, and highly complex TME [12–15]. Tumor tissues often exhibit hypoxic and acidic regions, a dense extracellular matrix, and overactive drug efflux pumps, all of which impede NP penetration and diminish therapeutic efficacy [16–18]. These barriers have driven the development of nanocarriers engineered with targeted, adaptive designs to overcome them. Modern nanocarriers incorporate both passive and active targeting strategies to enhance tumor selectivity [8]. A broad range of platforms, including liposomes, polymeric NPs, micelles, dendrimers, exosomes, and inorganic systems, use molecular design to control size, surface charge, and ligand orientation [19]. Such refinements promote selective accumulation within the TME and facilitate receptor-mediated cellular uptake, ultimately improving the therapeutic index of anticancer agents.

Beyond these considerations, recent studies underscore the value of predictive modeling for anticipating intratumoral distribution. For example, de Oliveira et al [20]. applied a compartmental mathematical model to describe the time-dependent biodistribution of lipid nanoemulsions in tumor-bearing mice, distinguishing between the tumor periphery and hypoxic core as separate compartments. In this framework, the biodistribution was modeled using a two-compartment kinetic system, in which the concentrations of the drug/nanocarrier in the tumor periphery (C_np) and core (C_nc) were expressed as time-dependent exponential functions determined by transfer rate constants. This quantitative description not only provided a better fit to experimental biodistribution data but also offered predictive insight into how architectural barriers delay drug penetration into hypoxic tumor cores. Importantly, the framework also incorporated biodistribution in systemic organs such as the liver and lungs, providing a quantitative picture of how nanocarriers are distributed beyond the primary tumor.

**Figure 1:**
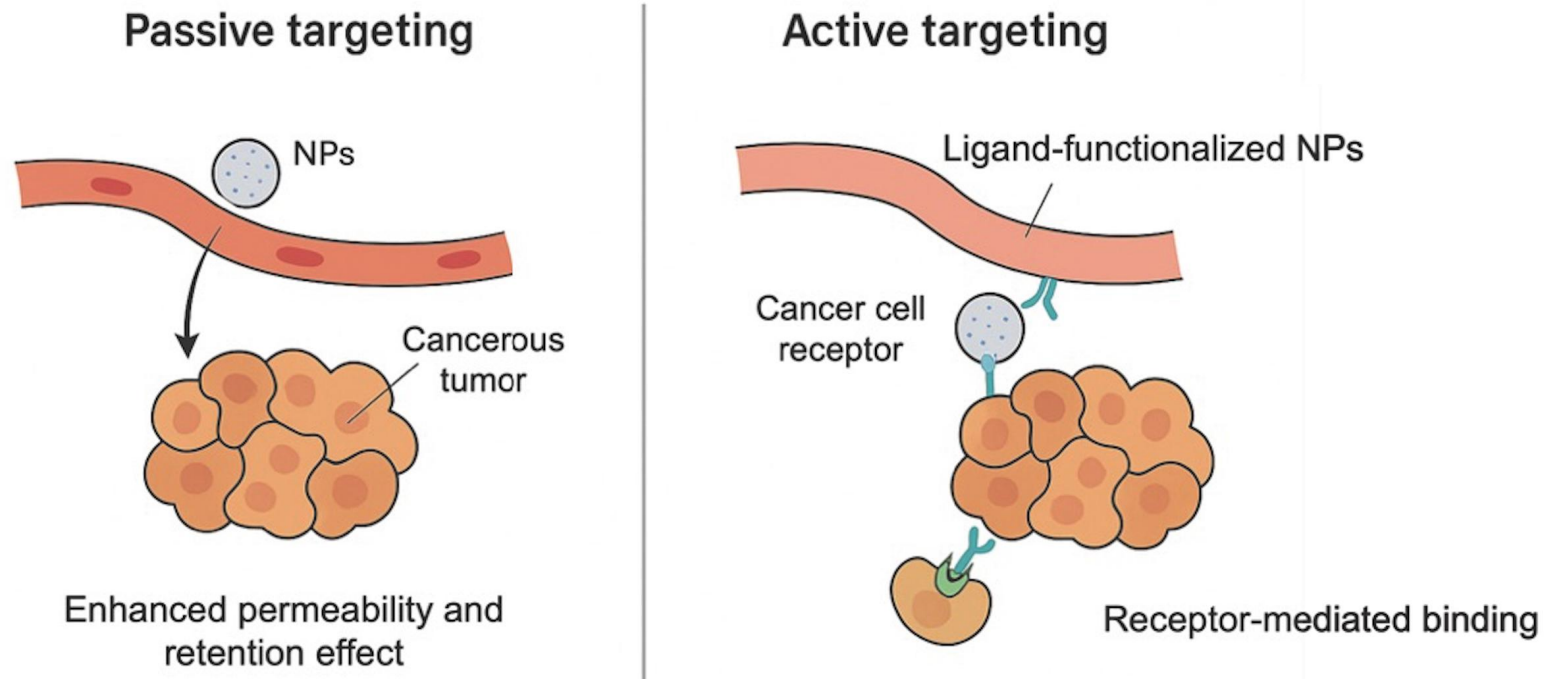
**Schematic comparison of passive and active targeting strategies for nanoparticle-based drug delivery in lung cancer.** NPs: nanoparticles.

The delivery systems are generally classified into two major categories: passive targeting, which exploits the enhanced permeability and retention (EPR) effect in leaky tumor vasculature, and active targeting, which utilizes ligand-specific interactions with tumor-associated markers [21]. Figure 1 summarizes and contrasts these two strategies, highlighting their underlying mechanisms and distinctive structural features.

### Passive targeting and the EPR effect

Molecularly optimized NPs (< 200 nm) exploit abnormal tumor vasculature via the EPR effect. This passive targeting relies on the NP’s physicochemical properties, which govern biodistribution and intratumoral retention. Lung tumors, like many solid tumors, often have disorganized vasculature with fenestrations that allow NPs to extravasate into the tumor interstitium. Meanwhile, defective lymphatic drainage in tumors leads to retention of NPs once they extravasate [22]. This passive accumulation mechanism enables significantly higher local drug concentrations in the tumor relative to free-drug formulations [23]. For example, liposomal doxorubicin and albumin-bound paclitaxel (nab-paclitaxel) both utilize the EPR effect to deliver greater amounts of drug into tumors while reducing systemic exposure [8]. In preclinical non-small-cell lung cancer (NSCLC) models, such NP formulations achieved improved tumor cell killing while largely sparing healthy cells.

However, the EPR effect alone is often insufficient; clinical studies show wide inter-patient variability in EPR-mediated accumulation [24]. Thus, while passive targeting via EPR is a foundational principle of nanomedicine, most modern designs also incorporate active targeting features to enhance specificity. pH-sensitive liposomes, for example, remain stable at physiological pH but release payloads in the acidic TME [25].

### Active targeting strategies

Active targeting decorates NPs with ligands that bind receptors or antigens overexpressed on cancer cells. In lung cancer, such targets include epidermal growth factor receptor (*EGFR*), transferrin receptors, folate receptors, integrins, and prostate-specific membrane antigen (PSMA). Conjugation of monoclonal antibodies, peptides, or other ligands enables receptor-mediated recognition and cellular uptake [26].

In addition to peptide- and antibody-based ligands, small-molecule modifiers can impart tissue-specific tropism. Notably, dimercaptosuccinic acid (DMSA) is a biocompatible coating that confers strong lung tropism. Biodistribution analyses revealed that DMSA-functionalized iron oxide NPs preferentially accumulated in pulmonary tissue shortly after intravenous administration, even prior to uptake in the liver or spleen [27]. This behavior supports DMSA as a potential ‘lung zip code’ ligand. Amaral et al. [28] provided an early demonstration of this lung-specific targeting strategy using a DMSA-functionalized polymeric nanocarrier. In their study, poly lactic-co-glycolic acid (PLGA) NPs coated with DMSA were loaded with amphotericin B, forming Nano-D-AMB, a DMSA-coated PLGA NP formulation of amphotericin B, to facilitate drug delivery to lung tissue. This DMSA-coated nanocarrier demonstrated therapeutic efficacy comparable to that of free amphotericin B, while significantly reducing systemic toxicity in vivo. Treated mice exhibited minimal weight loss, no signs of organ damage or stress, and even tolerated extended dosing intervals with fewer side effects than the conventional formulation. These outcomes underscore how functionalizing a nanocarrier with a lung-tropic molecule like DMSA can improve the therapeutic index of a payload by enhancing pulmonary targeting and reducing off-target toxicity.

Beyond small-molecule surface modifications, researchers have developed *EGFR*-targeted NPs for delivering small interfering RNA (siRNA) or chemotherapeutics to *EGFR*-mutant lung cancer cells [29]. For example, Ezhilarasan et al. designed gelatin NPs decorated with a biotinylated EGF that specifically binds to *EGFR* on lung cancer cells, resulting in enhanced NP internalization [26]. Moreover, transferrin-coated NPs that exploit transferrin receptor overexpression in lung tumors [30]. These targeted nanosystems have demonstrated higher accumulation on the surfaces of lung tumor cells and greater internalization into the cells compared to their non-targeted counterparts.

Active targeting can also address TME components, not only cancer cells. For instance, certain peptides can bind to fibroblast activation protein (*FAP*) on cancer-associated fibroblasts [31], or mannose can be used to target tumor-associated macrophages (TAMs), reprogramming TAMs from the immunosuppressive M2 state to the pro-inflammatory M1 state [32]. Such strategies help normalize the TME and improve therapy. Another promising avenue is targeting metastatic niches, although this is still under exploration. It is important to note that active targeting ligands must be carefully selected and designed. They increase the complexity and cost of NP formulations and must remain accessible on the NP surface. Additionally, heterogeneity in target expression can limit efficacy. Nonetheless, the combination of passive and active targeting has produced a new generation of nanomedicines with far greater tumor specificity. For lung cancer patients, this translates into potential treatments that concentrate on tumor cells or lung lesions while sparing healthy tissues, thereby reducing side effects [5].

## Nanocarrier platforms in lung cancer: established and emerging

The development of nanocarrier-based therapeutics has significantly transformed the treatment landscape for lung cancer. A diverse array of NP platforms has been engineered to address challenges such as inadequate tumor penetration, high systemic toxicity, and therapeutic resistance.

### Inhalable nanocarriers

Inhalable delivery systems represent a promising approach to achieve localized administration directly to the lungs, offering high drug concentrations at the tumor site while reducing systemic toxicity. For inhalation, carriers are formulated to achieve an aerodynamic diameter of 1–5 μm, despite their nanoscale physical size. Aerodynamic diameter dictates behavior in air and deposition within the respiratory tract [33]. In other words, while the carriers are nanoscale in physical dimensions, they can be formulated as porous or aggregated structures, or produced through spray-drying into inhalable microparticles, thereby exhibiting an effective aerodynamic diameter in the 1–5 μm range [34]. This preserves nanoscale advantages while enabling deep alveolar deposition. A variety of nanocarrier platforms have been engineered for inhalation, each leveraging distinct design features to enhance lung cancer therapy [35–37].

However, a critical limitation of inhaled nanocarriers is ensuring their efficient deposition in the alveoli and subsequent penetration into tumor tissue, as incomplete distal lung delivery or rapid clearance can compromise therapeutic efficacy [23]. Clinically relevant aerosol devices now help address these challenges. For example, Fu et al. [38] developed an inhalable nanoliposome co-loaded with the *EGFR* inhibitor osimertinib and a DNA plasmid targeting insulin-like growth factor 2 mRNA-binding protein 3 (*IGF2BP3*), administered via a vibrating mesh nebulizer. This system achieved deep alveolar deposition and efficient uptake by tumor cells in mice, resulting in the suppression of both primary lung tumors and brain metastases, enhanced antitumor immunity, and significantly prolonged survival compared to conventional treatment.

**Figure 2:**
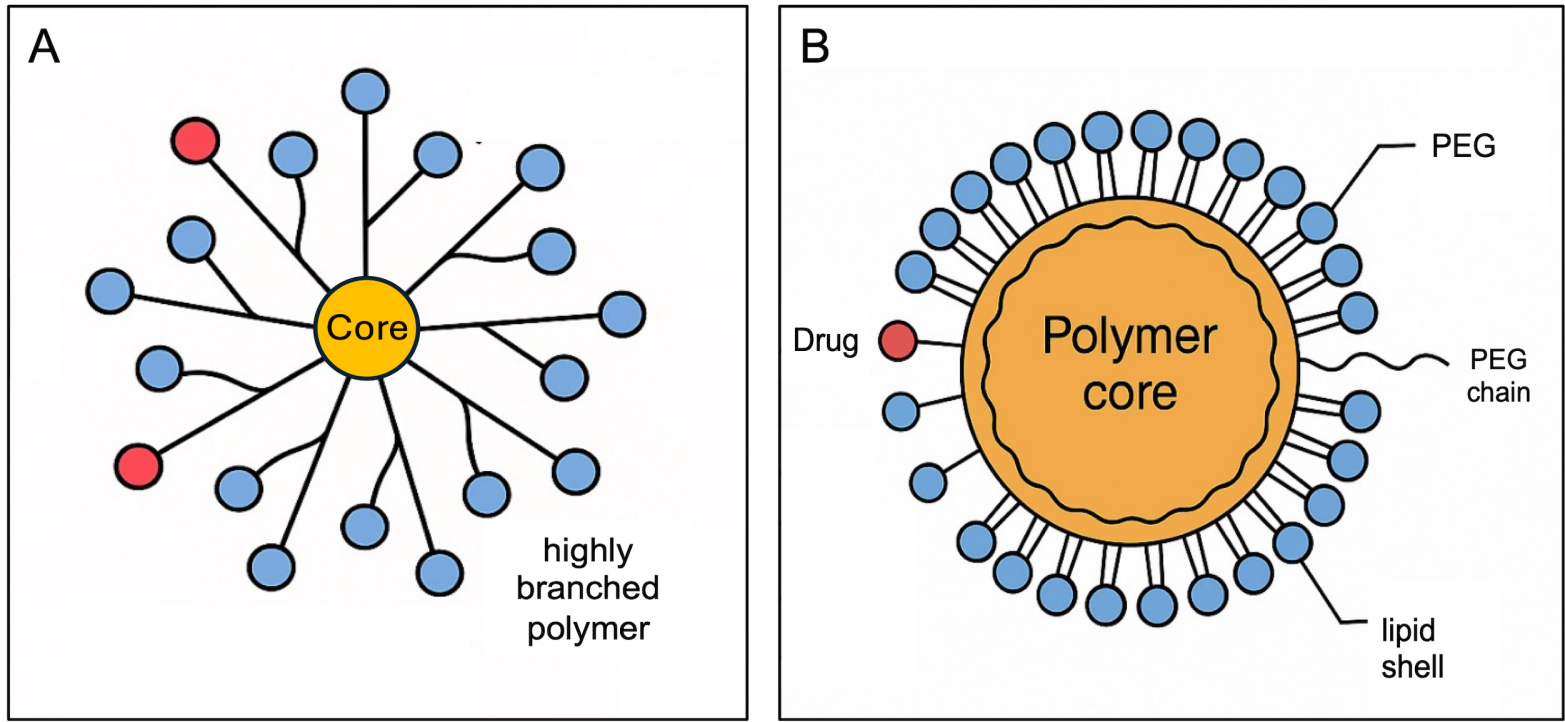
**Schematic illustration of nanoparticles.** (**A**) Dendrimer-based nanoparticle; (**B**) polymer-lipid hybrid nanoparticle. PEG: polyethylene glycol.

Beyond these considerations, dendrimer-based NPs exemplify this approach, as their highly branched polymer architecture enables high drug payloads and multivalent targeting functionality (Figure 2A). For instance, a cationic poly(amidoamine) dendrimer loaded with the *EGFR* inhibitor erlotinib demonstrated significantly greater selective toxicity toward NSCLC cells compared to the free drug, highlighting the potential of dendrimers for targeted, sustained-release inhalable therapy [39]. Polymer-lipid hybrids and polyethylene glycol (PEG)-coated (PEGylated) polymeric NPs have been adapted for inhalation to co-deliver agents such as chemotherapy with a gene or siRNA (Figure 2B). These hybrid systems exhibit extended pulmonary residence times and enhanced mucosal penetration, translating into higher intratumoral drug concentrations in the lung [36, 40].

**Figure 3:**
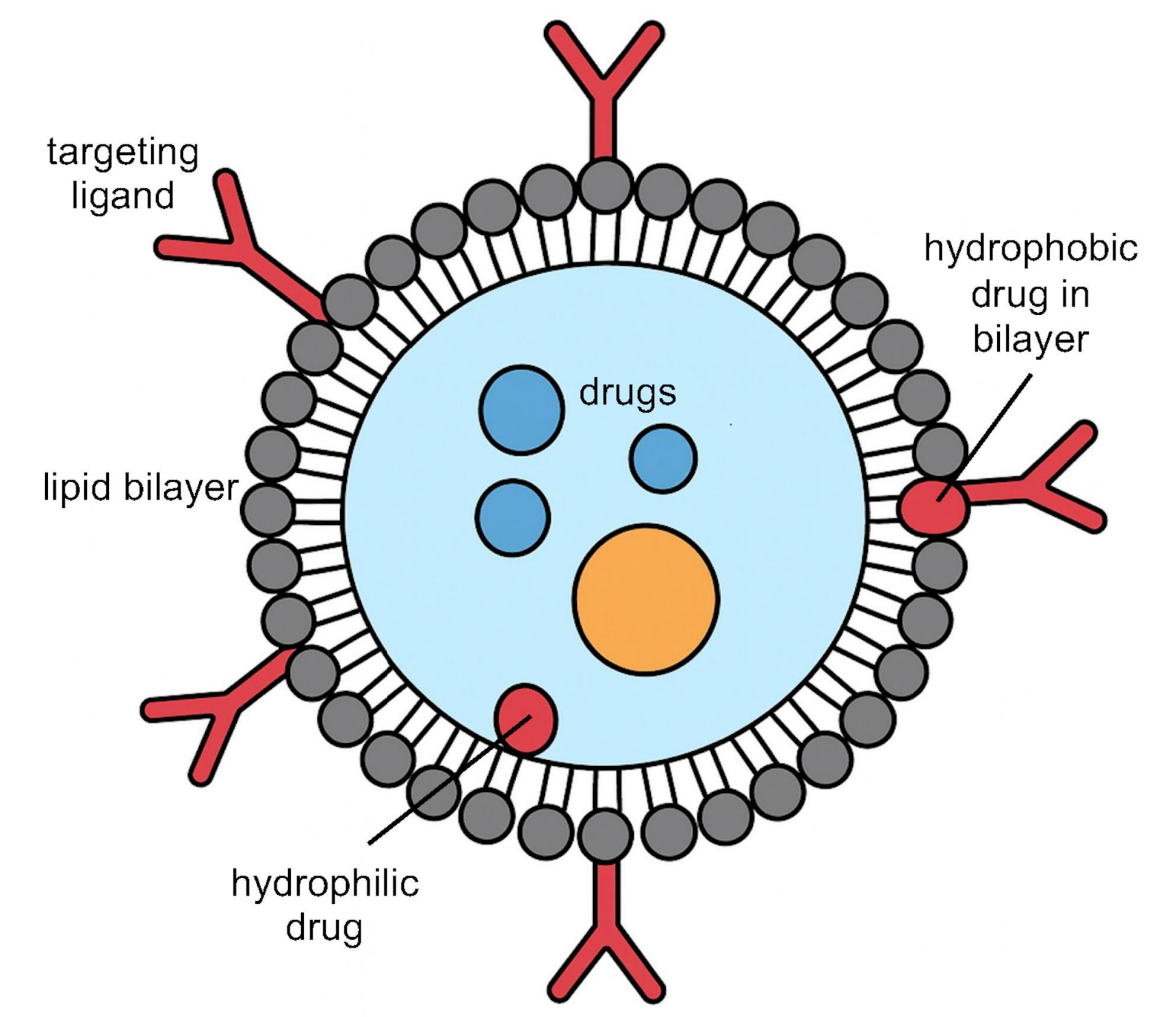
Schematic illustration of active targeted liposomes.

Active targeted liposomes represent another inhalable nanocarrier strategy (Figure 3). These are immunoliposomes functionalized with ligands that actively bind to cancer cell receptors, thereby improving selective uptake. In one study, an inhaled anti-EGFR immunoliposome delivering the *EGFR* tyrosine kinase inhibitor osimertinib achieved significantly greater anti-tumor efficacy than a non-targeted liposomal formulation, as evidenced by enhanced cytotoxic potency and suppression of tumor cell migration in *EGFR*-mutant lung cancer models [41].

**Figure 4:**
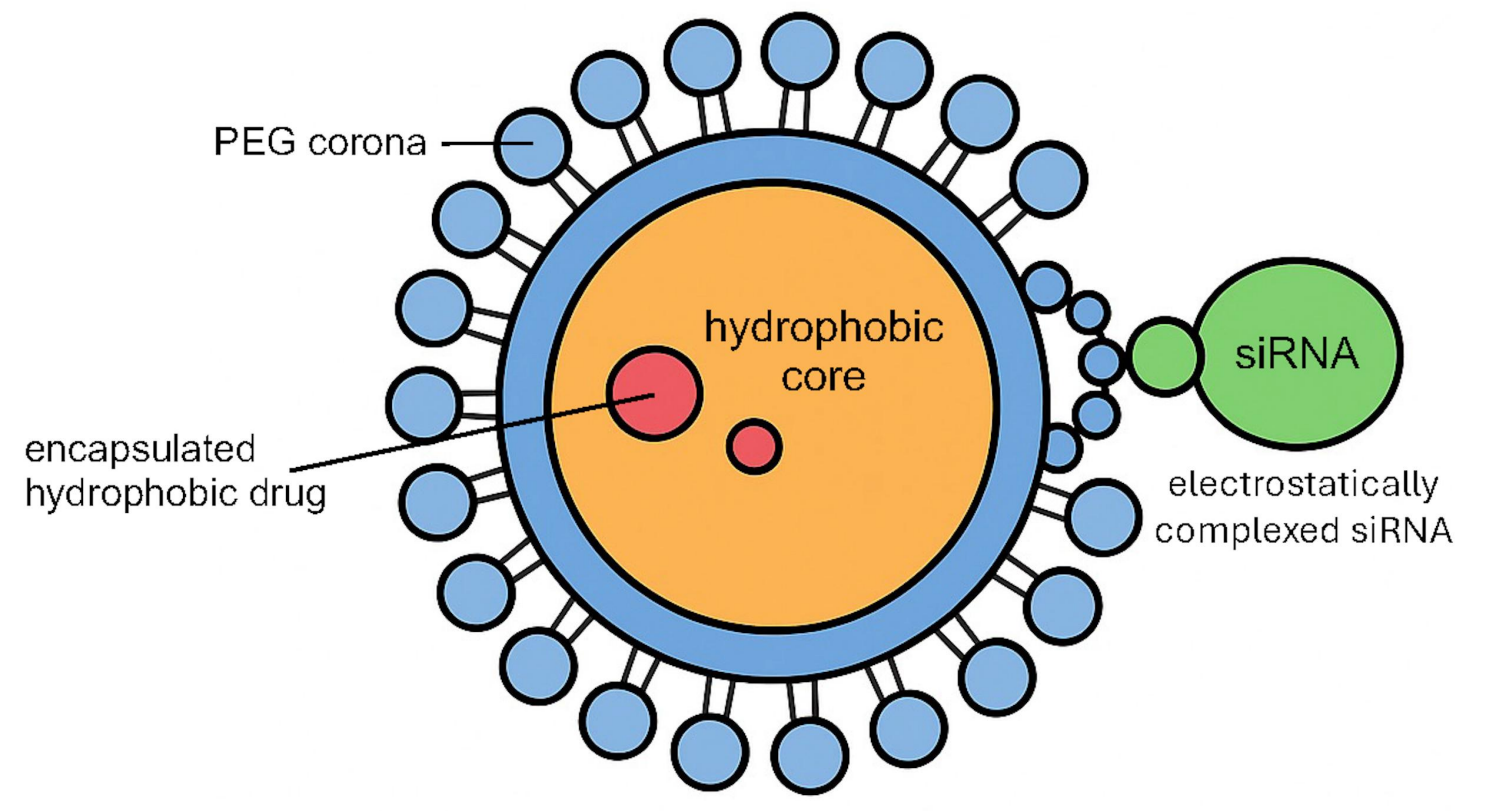
**Schematic illustration of the polymeric micelle loaded with siRNA.** PEG: polyethylene glycol; siRNA: small interfering RNA.

Beyond conventional drugs, inhalable nanocarriers have been developed to deliver nucleic acids. For example, micelles loaded with siRNA can be administered as an aerosol to ferry siRNAs into lung tumors, silencing oncogenic or drug-resistance genes at their source (Figure 4). Notably, a nebulized polymeric NP carrying siRNAs against βIII-tubulin and polo-like kinase 1 (*PLK1*) effectively accumulated in an orthotopic lung tumor model, knocked down these target genes, and significantly delayed tumor progression [42]. Such gene-silencing inhalable micelles, especially when combined with chemotherapy, have demonstrated a capacity to overcome resistance mechanisms and produce synergistic tumor inhibition in preclinical studies.

Furthermore, stimulus-responsive designs have been integrated into inhalable formats. One example is an enzyme-responsive “nanoreactor” approach in which a NP is coated with a gelatin-glutamate shell that remains intact during aerosol delivery but is specifically degraded by matrix metalloproteinase-2 (MMP-2) in the lung TME. Upon this enzymatic trigger, the inhalable nanoreactor releases a ferrocene payload that catalyzes the production of a burst of reactive oxygen species (ROS) within the tumor, inducing ferroptotic cell death in cancer cells [43]. This smart design achieved over double the lung drug accumulation and markedly enhanced tumor suppression in an orthotopic lung cancer model, underscoring the efficacy of TME-triggered inhalable therapy. Ultimately, biomimetic surfactant-mimicking NPs provide a strategy to enhance lung retention. By incorporating lung surfactant lipids or peptides into their formulation, the inhalable NPs assimilate into the alveolar lining fluid and evade rapid clearance by alveolar macrophages. This surfactant-coated design prolongs the residence time of the therapeutic carrier in pulmonary tissue and has been shown to enhance local drug bioavailability and anti-tumor effects in the lungs [44].

### Exosome-based drug delivery systems

**Figure 5:**
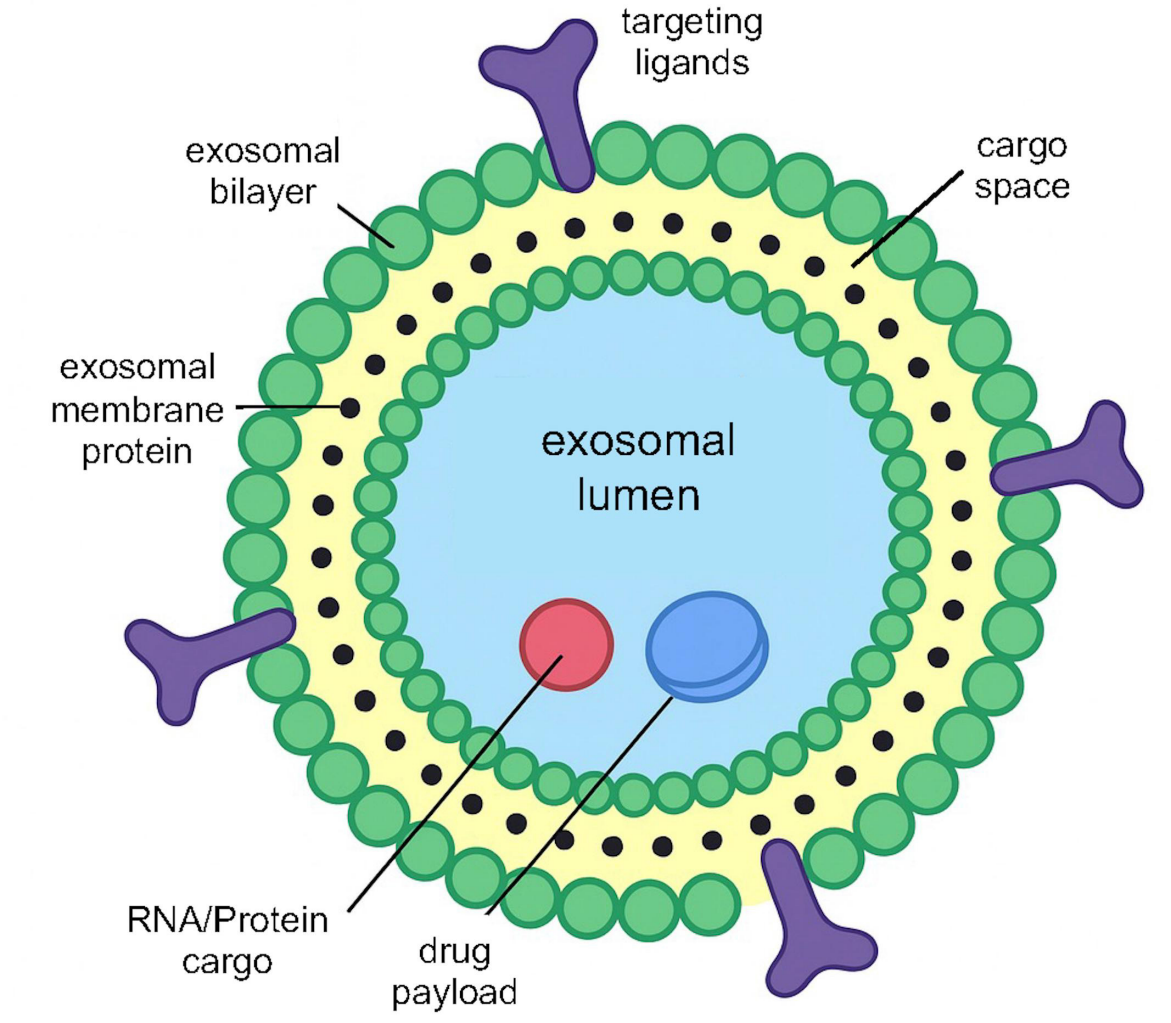
Schematic illustration of exosome structural features.

Naturally derived vesicles such as exosomes leverage endogenous targeting and low immunogenicity as delivery vehicles. Exosomes are naturally occurring extracellular vesicles (30–150 nm in size) released by cells as intercellular messengers, possessing several features advantageous for drug delivery, including innate biocompatibility, the ability to cross biological barriers, and an intrinsic homing capacity for specific target tissues [45]. In lung cancer therapy, researchers are repurposing exosomes to carry anticancer payloads, including chemotherapeutic drugs, siRNA, mRNA, or even clustered regularly interspaced short palindromic repeats (CRISPR) and CRISPR-associated protein 9 (Cas9) (CRISPR-Cas9) components (Figure 5). A key advantage is minimal immunogenicity. Because they are derived from the body’s own cells, exosomes can often evade immune surveillance more effectively than synthetic NPs. For example, inhaled exosomes elicit less pulmonary inflammation than many synthetic lipid NPs [46]. This property makes exosomes a compelling platform for overcoming biological barriers and potentially targeting metastatic niches. Exosomes have even been shown to cross the blood-brain barrier, raising the possibility of delivering therapies to lung cancer brain metastases [47]. While exosome-based drug delivery for lung cancer is still in the experimental stage, progress in recent years has been rapid. Challenges, including scalable production and efficient loading, are being actively addressed through techniques like electroporation, membrane fusion, and genetic engineering of exosome-producing cells [45]. Early-phase clinical trials of exosome therapeutics are already underway in other cancer types and disease areas, and trials for lung cancer are anticipated in the near future.

### Stimuli-responsive nanocarriers

Stimuli-responsive (‘smart’) nanocarriers constitute an advanced class of delivery systems. This approach enhances precision by transporting drugs in an inert manner and releasing them predominantly at tumor sites. In lung cancer, where the TME is characterized by features like acidic pH, elevated protease levels, abnormal redox conditions, and hypoxia, stimuli-responsive nanocarriers can exploit these differences to achieve on-demand drug release [48–50]. For example, pH-sensitive NPs remain stable at normal physiological pH but rapidly dissociate in the acidic milieu of tumor tissue or endosomes, thereby preferentially releasing their payload in cancer cells [48]. Likewise, enzyme-cleavable linkers confine release to enzyme-rich tumors [49]. Other designs include redox-responsive systems that break apart in high-glutathione (GSH) environments, as well as hypoxia-responsive carriers that release drugs under low-oxygen conditions [50]. By integrating such bio-responsive triggers, these nanocarriers minimize off-target drug release and toxicity, improving the therapeutic index of anticancer agents.

Recent research provides compelling evidence that stimuli-responsive nanocarriers can significantly enhance the efficacy of lung cancer treatment. One example is an enzyme-responsive “nanoreactor” reported by Wang et al. [43]. In this system, a lipid-based NP was coated with a gelatin-glutamate corona and co-encapsulated two agents: fluvastatin and ferrocene. Upon reaching the lung tumor site, the abundant MMP-2 enzymes in the TME degraded the gelatin shell, thereby triggering the release of ferrocene inside the tumor tissue. The liberated ferrocene catalyzed the excessive production of ROS directly at the tumor site, overwhelming the cancer cells’ antioxidant defenses and inducing ferroptotic cell death. This design leverages tumor-associated enzymes as triggers, producing highly localized cytotoxicity.

### Theranostic nanocarriers

Theranostic nanocarriers combine therapeutic and diagnostic functions in a single platform. This dual capability directly tackles a major oncology challenge: the lack of real-time treatment monitoring and personalization in current practice [51]. Typically, therapy is administered, and tumor response is evaluated only weeks later via separate imaging modalities, which delays critical adjustments. By contrast, theranostic NPs deliver treatment while concurrently enabling imaging of drug distribution and tumor response [52]. Real-time feedback enables earlier treatment modification and better patient stratification. To achieve this, researchers engineer NPs to carry both a therapeutic payload and an imaging agent within the same construct [53, 54]. The imaging component may be a contrast agent for magnetic resonance imaging (MRI), a positron emission tomography/single photon emission computed tomography (PET/SPECT) radiotracer, or a fluorescent probe, while the therapeutic payload can range from conventional chemotherapeutics to gene-silencing molecules or even radioisotopes. Notably, they selectively accumulate in lung tumors, enabling non-invasive visualization of delivery in vivo [54, 55].

Recent studies highlight the promising therapeutic potential of theranostic nanocarriers in lung cancer, as well as their imminent clinical relevance. For example, Qiu et al. [54] developed a pH-sensitive core-shell NP (AHA@MnP/QCT) loaded with the natural drug quercetin and doped with Mn^2+^ ions for built-in MRI contrast. In lung cancer models, this theranostic NP preferentially targeted NSCLC tumors, inducing ferroptosis and apoptosis in cancer cells, resulting in significant tumor growth inhibition. At the same time, the Mn^2+^ provided T2-weighted MRI visibility, confirming that the NPs effectively reached and penetrated tumor sites. Similarly, Lu et al. [55] reported an “all-in-one” theranostic nanosystem based on a zeolite imidazole framework-8 (ZIF-8) metal-organic framework co-encapsulating the chemotherapy drug paclitaxel and the near-infrared (NIR) dye indocyanine green. This dual-responsive nanocarrier enabled simultaneous NIR imaging and photothermal therapy: upon NIR laser irradiation, the NP generated heat and triggered on-demand release of paclitaxel in the acidic TME. In NSCLC models, the combination of NIR-guided photothermal ablation with localized chemotherapy produced synergistic tumor cell killing and significantly suppressed tumor growth compared to either modality alone.

An example of such multifunctionality is provided by DMSA-stabilized magnetic NPs, which combine diagnostic and therapeutic modalities in a single platform. These nanostructures offer strong T2-weighted magnetic resonance contrast, while also serving as heating agents for magnetic hyperthermia when exposed to an alternating magnetic field [56]. Importantly, their biodistribution studies confirmed preferential deposition in the lungs, not only in murine models but also in nonhuman primates, establishing robust preclinical evidence of pulmonary tropism. Such theranostic versatility highlights the potential of DMSA-coated magnetic NPs as a clinically relevant platform for lung cancer imaging and site-specific ablation. These examples demonstrate the breadth of nanocarrier-based theranostics, from stimuli-responsive polymers to lung-targeted magnetic systems.

Theranostic nanocarriers are now rapidly moving toward clinical translation. They align with personalized medicine initiatives and support adaptive care. Notably, some theranostic agents, such as iron oxide NPs used for MRI-visible thermal ablation, have already entered clinical trials for other cancer types, and similar concepts are being explored for lung tumors [57]. Achieving regulatory approval for theranostic nanomedicines will require demonstrating not only therapeutic safety and efficacy but also the reliability of the diagnostic component. Nevertheless, recent preclinical successes in lung cancer are encouraging, marking a paradigm shift in which nanocarriers evolve from passive drug delivery vehicles to active participants in both treating and tracking disease.

## Nanotechnology in combination with immunotherapy

Immunotherapy has transformed lung cancer care, yet only a fraction of patients achieve durable responses. Challenges such as tumor immune evasion, the systemic toxicity of immunostimulatory drugs, and an immunosuppressive TME limit broader success [46]. Engineered nanocarriers can localize and amplify immune-modulating agents in tumors, enhancing efficacy while minimizing off-target effects. Recent progress between 2022 and 2025 has led to the development of various nano-immunotherapy strategies that are now progressing toward clinical application, including NP-enabled checkpoint inhibition, nanovaccine-based immune priming, and TME-modulating nanotherapies.

### Nanoparticle-based immune checkpoint modulation

Nanoscale delivery systems are being harnessed to improve and localize checkpoint blockade therapy. One approach co-delivers checkpoint inhibitors with synergistic agents in a single formulation. For example, Reda et al. [58] developed a polymer-coated silica NP co-loaded with a programmed death-ligand 1 (PD-L1) antibody and a *PLK1* inhibitor. This nanotherapy achieved potent lung tumor regression in mice, using five-fold lower doses of each drug, while simultaneously inducing cancer cell death and blocking adaptive immune resistance. Tumor-targeted delivery reduced the systemic toxicity observed with free *PLK1* inhibitors or cytokine therapies. Another tactic uses multifunctional nanosystems to block checkpoint pathways in situ, replacing or augmenting antibodies. Song et al. [59] reported an all-synthetic polymeric NP that provides programmed cell death protein 1 (PD-1)/PD-L1 checkpoint blockade together with localized tumor cell killing. Their NP carried a PD-L1 antagonist on its surface and an inducer of immunogenic cell death (ICD). Upon reaching the tumor, this construct bound and downregulated PD-L1 on cancer cells while releasing doxorubicin to induce ICD. In mouse models, the nano-formulation triggered robust antitumor T-cell responses and achieved complete tumor regressions in ~60% of treated animals, significantly superior to the outcomes of either agent alone, illustrating the cooperative benefit of co-delivery. Similarly, other nano-constructs integrate checkpoint inhibitors with modalities like photothermal therapy to convert tumors into in situ vaccines while blocking immune escape. Yu et al. [60] demonstrated a polymer NP that, under NIR irradiation, ablated primary tumors and simultaneously released PD-1/PD-L1 blocking agents, suppressing distant lung tumor growth by combining ICD-induced systemic immunity with checkpoint blockade. Overall, NP-based checkpoint modulation concentrates immunotherapy within tumors and limits systemic exposure. Early-phase trials are beginning to explore such combinations in lung cancer patients, reflecting a clear path from nano-engineered concepts toward clinical translation.

### Nanovaccine platforms for anticancer immunity

Nanotechnology reinvigorates cancer vaccines by co-delivering antigens and adjuvants to amplify cytotoxic T-cell priming. Novel “nanovaccine” platforms use NPs to co-deliver tumor antigens and immune adjuvants in a targeted fashion, thereby amplifying cytotoxic T-lymphocyte priming. Baljon et al. [61] reported a versatile NP vaccine that encapsulates personalized peptide neoantigens together with synergistic adjuvants. The pH-responsive polymer vesicles ensured co-release of antigen and adjuvants in the same dendritic cell, dramatically enhancing antigen presentation and T-cell activation compared to free peptide vaccines. In multiple mouse tumor models, this nanovaccine induced robust CD8^+^ T-cell responses, also known as cytotoxic T lymphocytes, which slowed tumor growth and improved response rates to anti-PD-1 therapy. The formulation is also scalable, underscoring its translational potential. To complement synthetic nanovaccines, biomimetic approaches have emerged. For example, ICD-tumor cell membrane-coated NPs loaded with cytosine guanosine dinucleotide (CpG) have demonstrated the ability to trigger robust dendritic cell maturation and T-cell infiltration [62]. By combining intrinsic tumor antigens with potent immune stimulation, these biomimetic nanovaccines offer a promising strategy to enhance immunogenicity, particularly in tumor types that are poorly responsive to immunotherapy, such as lung cancer. Their ability to activate innate and adaptive immunity simultaneously highlights their relevance as next-generation cancer vaccine platforms. Li et al. [63] developed a personalized nanovaccine by enriching cancer cell membranes with neoantigens and coupling them with a stimulator of interferon genes (STING)-activating polymer adjuvant. These membrane-coated NPs elicited polyclonal T-cell responses, resulting in the regression of lung tumors and inhibition of metastases. The approach generated robust CD8^+^ memory and elicited a superior T-cell response compared to earlier vaccines.

### Nanocarriers for tumor microenvironment reprogramming

Nanocarriers can reprogram TAMs from an immunosuppressive M2 phenotype to an antitumor M1 phenotype. For example, superparamagnetic iron oxide nanoparticles (SPIONs) formulated as polymeric micelles have been shown to repolarize TAMs and reshape the lung TME. In an anaplastic lymphoma kinase (*ALK*)-positive lung cancer model, intratracheal instillation of SPION-containing micelles not only delayed tumor growth but also, when administered after first-line *ALK* tyrosine kinase inhibitor therapy, halted the regrowth of resistant tumors, effectively preventing relapse [64]. Similarly, delivering immunogenic or inflammatory payloads with NPs can “re-educate” TAMs to attack the tumor. Zhao et al. [65] demonstrated that a nanodiamond-doxorubicin conjugate, combined with PD-L1 checkpoint blockade therapy, synergistically shifted TAMs to a tumoricidal state, turning the macrophages against lung tumor cells and enhancing overall antitumor efficacy.

Beyond targeting TAMs, nanocarriers are being used to deliver innate immune agonists and other immunomodulatory payloads directly to lung tumors, thereby overcoming immunosuppression. For instance, NPs loaded with cyclic dinucleotide STING agonists have achieved potent immune activation in the lung. In a recent study, an inhalable STING nanocarrier markedly attenuated the immunosuppressive TME and significantly improved responses to PD-1/PD-L1 checkpoint blockade in an NSCLC model [66]. Other NP platforms carry immunostimulatory agents such as Toll-like receptor agonists or deliver siRNAs/small-molecule inhibitors that block immunosuppressive pathways in the TME to enhance immune checkpoint inhibitor therapy. For example, a macrophage membrane-coated NP encapsulating a transforming growth factor-β (TGF-β) receptor 1 inhibitor was shown to enhance anti-PD-1 therapy in lung cancer models, as it prevented TAMs from acquiring the M2 phenotype, curtailed tumor metastasis, and expanded cytotoxic T cells, thereby significantly improving the effectiveness of checkpoint inhibitors [67]. These multipronged nanomedicine approaches are actively reprogramming the lung TME and have demonstrated enhanced responsiveness to immunotherapy in preclinical models.

## Strategies to overcome drug resistance in lung cancer

Intrinsic and acquired resistance remain formidable obstacles. Nanotechnology has emerged as a promising modality to counteract these resistance mechanisms by enabling multifaceted interventions such as altered pharmacokinetics, co-delivery of therapeutic agents, and modulation of intracellular drug fate. This section discusses the mechanistic basis and translational outlook of nano-enabled strategies to overcome resistance in lung cancer, while clinical validation is addressed in the subsequent section on Translation and clinical trials.

One promising approach involves the induction of ferroptosis, a regulated, iron-dependent form of cell death distinct from apoptosis, via nanomaterial-based systems. Ferroptosis involves the accumulation of lipid peroxides and ROS, which can eliminate apoptosis-resistant tumor cells. Nanocarriers have been designed to deliver iron donors, GSH-depleting agents, or metabolic modulators to induce ferroptotic cell death [68]. For instance, Wang et al. [69] developed a chondroitin sulfate-based NP co-encapsulating curcumin and cinnamaldehyde, which elevated intracellular ROS and depleted GSH, culminating in the inhibition of GSH peroxidase 4 (*GPX4*) and effective induction of ferroptosis in paclitaxel- and cisplatin-resistant NSCLC models. Gene editing, particularly CRISPR-Cas9, offers a route to overcoming resistance by silencing or correcting the drivers of resistance. This includes genes involved in drug efflux, anti-apoptotic signaling, or mutations that confer resistance [70]. The main barrier to clinical translation lies in the delivery of CRISPR components to solid tumors. Nanocarrier systems have been developed to overcome this challenge by facilitating the targeted delivery of genome-editing machinery into cells. A notable example is the study by Liu et al. [46], in which CRISPR-Cas9 complexes targeting cyclin-dependent kinase 4 (*CDK4*) were encapsulated in biomimetic NPs derived from cryo-shocked lung tumor cell membranes, thus enabling homotypic targeting. When administered to Kirsten rat sarcoma viral oncogene homolog (*KRAS*)-mutant lung tumor-bearing mice, these NPs achieved precise in vivo *CDK4* knockout, resulting in substantial tumor regression and extended survival. Building upon this concept, other studies are exploring lipid-based nanocarriers and exosome-mimetic vesicles, engineered for responsiveness to pH, redox states, or enzymatic cues in the TME. Although still in early development, these platforms represent a high-potential strategy for reversing resistance through genetic modulation [71].

Reformulating established chemotherapies into NP systems can bypass conventional resistance mechanisms. Liposomal irinotecan (NALIRI; Onivyde), polymeric micellar paclitaxel (Pm-Pac; Genexol-PM), and nab-paclitaxel exemplify such innovations. These nanoformulations enhance drug stability, improve tumor penetration, and reduce systemic toxicity [72]. Pm-Pac, by eliminating toxic solvents, improves safety and bioavailability [73], while nab-paclitaxel leverages albumin-mediated transcytosis to facilitate deep tumor access [74].

Finally, antibody-drug conjugates (ADCs) offer tumor-selective delivery of cytotoxic payloads, representing a powerful tool to address resistance. In NSCLC, the antigens trophoblast cell surface protein 2 (*TROP2*) and human *EGFR 3* (*HER3*) have become critical targets for ADCs, especially in tumors exhibiting resistance to *EGFR* tyrosine kinase inhibitors (TKIs) or chemotherapy relapse. The *TROP2*-directed ADC datopotamab deruxtecan (Dato-DXd) delivers a topoisomerase I inhibitor specifically to *TROP2^+^* tumor cells, minimizing off-target toxicity [75]. Patritumab deruxtecan (*HER3*-DXd) targets tumors with *EGFR* mutations that develop *HER3*-driven resistance mechanisms [76, 77]. These ADCs bypass efflux-mediated resistance and facilitate intracellular drug accumulation in poorly perfused or heterogeneous regions [78]. Moreover, the selective targeting afforded by antibody-based systems permits the use of highly potent cytotoxins that would otherwise be intolerable, improving the therapeutic index [79]. Table 1 summarizes additional representative examples of experimental and early clinical evidence supporting each strategy.

**Table 1:** Representative nanotechnology strategies to overcome drug resistance in lung cancer.

**Strategy**	**Nanoplatform**	**Model**	**Study type**	**Key outcome**	**Reference**
**Ferroptosis-inducing**	Ball-rod Janus NP (Fe^3+^/GOx/Sorafenib)	A549 NSCLC xenograft	Preclinical	*GPX4* suppression + ROS ↑ → tumor inhibition in sorafenib-resistant lung tumors	[80]
	FePt@HA NP (targeting CD44)	*EGFR*-TKI-resistant mesenchymal NSCLC	Preclinical	Ferroptosis in erlotinib-resistant EMT-NSCLC cells	[81]
**CRISPR via nanocarriers**	HA liposome/protamine + Cas9-*MTH1*	NSCLC (A549); liver metastasis mice	Preclinical	*MTH1* knockout → reduced lung tumor + liver mets	[82]
	C14-PEG-PEI micelleplex (Cas9-*KRAS* G12S)	*KRAS*-mutant NSCLC (A549)	Preclinical	*KRAS* editing → cell death ↑, migration ↓	[83]
	Cryo-shocked tumor vesicle (Cas9-*CDK4*)	*KRAS*-driven NSCLC mice	Preclinical	*CDK4* knockout → regression + survival benefit	[84]
**Reformulated nano-chemotherapy**	Inhalable liposome (osimertinib + *IGF2BP3*-plasmid)	*EGFR* ^+^ NSCLC + brain mets (mouse)	Preclinical	Dual lung/brain targeting → tumor & brain mets suppression	[85]
	PEG-polymer NP (cisplatin + fluvastatin)	*TP53*-mutant NSCLC (H1975)	Preclinical	Mutant p53 degradation → restored cisplatin sensitivity	[86]
	Cetuximab-chitosan NP (PTX + quercetin)	A549/Taxol-resistant model	Preclinical	P-gp bypassed → paclitaxel re-sensitization	[87]
**ADCs**	Telisotuzumab vedotin (anti-c-MET-MMAE)	*EGFR*-TKI-resistant NSCLC (high c-MET)	Early clinical	ORR 50% + PFS benefit with osimertinib in TKI-resistant c-MET + NSCLC	[88]
	DB-1314 (anti-DLL3-topoisomerase inhibitor)	Drug-resistant SCLC (PDX & cell lines)	Preclinical	DLL3 ADC reversed platinum resistance, durable tumor regression	[89]
	Tusamitamab ravtansine (anti-CEACAM5-DM4)	Heavily pretreated CEACAM5-high NSCLC	Early clinical	ORR ~20%, durable responses in multi-drug-resistant adenocarcinoma	[90]

NP: nanoparticle; Gox: glucose oxidase; Fe^3+^: ferric ion; ROS: reactive oxygen species; *GPX4*: glutathione peroxidase 4; NSCLC: non-small cell lung cancer; *EGFR*-TKI: epidermal growth factor receptor-tyrosine kinase inhibitor; EMT: epithelial-mesenchymal transition; CRISPR: clustered regularly interspaced short palindromic repeats; HA: hyaluronic acid; *MTH1*: MutT homolog 1; PEG: polyethylene glycol; PEI: polyethylenimine; *KRAS*: Kirsten rat sarcoma viral oncogene; *CDK4*: cyclin-dependent kinase 4; *IGF2BP3*: insulin-like growth factor 2 mRNA-binding protein 3; PTX: paclitaxel; P-gp: P-glycoprotein; ADCs: antibody-drug conjugates; MET: mesenchymal-epithelial transition; MMAE: monomethyl auristatin E; ORR: objective response rate; PFS: progression-free survival; DLL3: delta-like ligand 3; PDX: patient-derived xenograft; SCLC: small cell lung cancer; CEACAM5: carcinoembryonic antigen-related cell adhesion molecule 5; DM4: maytansinoid DM4.

## Translation and clinical trials

In recent years, numerous nanomedicine strategies have advanced into clinical trials. Between 2020 and 2025, a diverse range of nanotechnology-enabled interventions, spanning ADCs, NP vaccines, gene-loaded liposomes, and inhalable nanotherapeutics, entered clinical evaluation, offering new hope to patients with advanced or treatment-refractory disease. Key examples are summarized below.

### Targeted nanotherapies and ADCs in the clinic

Targeted nanocarriers, notably ADCs, offer tumor-selective payload delivery that improves therapeutic index. Clinically validated ADCs such as trastuzumab emtansine (T-DM1) and brentuximab vedotin laid the groundwork for next-generation platforms. Recent trials have highlighted several new ADCs in lung cancer, including Dato-DXd for *TROP2* [91], *HER3*-DXd for *EGFR*-resistant NSCLC [76, 92], and T-DXd [93]. T-DXd, previously approved for HER2-positive breast cancer, demonstrated over 50% response rates in HER2-mutant NSCLC, resulting in its first Food and Drug Administration (FDA) approval for lung cancer in 2022 [91, 93]. In addition, telisotuzumab vedotin (Teliso-V) demonstrated activity in mesenchymal-epithelial transition (MET)-overexpressing NSCLC [88], and disitamab vedotin (RC48) has shown potential in HER2-altered lung cancer [94]. By contrast, the delta-like ligand 3 (DLL3)-targeting ADC rovalpituzumab tesirine (Rova-T) failed to improve survival [95].

Beyond ADCs, novel constructs are advancing. For example, the *EGFR*-targeted bacterial nanocell (EDV^TM^), a biomimetic system, is currently under investigation in a phase I/IIa basket trial for *EGFR*-positive solid tumors, including NSCLC. This platform combines selective delivery of ultra-potent cytotoxins with immune stimulation and has shown favorable tolerability in early studies [96].

### Nanoparticle vaccines and radioenhancers

NP-based vaccines are advancing into clinical trials as platforms to elicit tumor-specific immunity. BNT116, an mRNA lipid NP vaccine encoding six NSCLC-associated antigens, demonstrated antigen-specific T-cell responses and acceptable safety in its first-in-human trial. This represents one of the first multivalent mRNA nanovaccine approaches in lung cancer [97, 98]. Meanwhile, radio sensitization using NP platforms is emerging as a strategy to improve local tumor control. NBTXR3, a first-in-class hafnium oxide NP radioenhancer injected intratumorally, amplifies the effects of radiotherapy [99, 100]. Initial trials in NSCLC demonstrated safety and feasibility, and more recent reports have described immune-mediated abscopal effects and clinical responses in unresectable NSCLC. These platforms support multimodal therapy, functioning as delivery vehicles, immune stimulators, and radiotherapy amplifiers.

### Chemotherapy and gene delivery platforms

NP formulations of cytotoxic drugs are enhancing delivery precision and reducing systemic toxicity. Clinical examples include nab-paclitaxel in advanced NSCLC [11], NALIRI [101], and Pm-Pac [73], all of which have demonstrated clinical benefit with improved safety profiles. Inhalable lipid-polymer hybrid NPs have also shown safe, lung-specific delivery in early trials [44]. Moreover, combination regimens using nab-paclitaxel, together with immune checkpoint inhibitors and anti-angiogenic agents, have yielded encouraging efficacy, achieving a progression-free survival of over 14 months in patients with metastatic adenocarcinoma [72]. Gene delivery approaches are also advancing: the NP-based gene therapy Oncoprex (REQORSA), which delivers the tumor-suppressor gene TUSC2, has demonstrated safety and early efficacy in NSCLC trials, including combinations with *EGFR* inhibitors or immunotherapy [102]. Similarly, CRISPR-edited PD-1 knockout T cells, administered to NSCLC patients, have shown the feasibility of NP-facilitated genome editing in a solid tumor setting [103]. These developments mark the entry of gene nanomedicine into lung cancer trials.

In summary, the 2020–2025 period has witnessed a significant expansion of nanotechnology’s clinical application in lung cancer, spanning next-generation ADCs, nanovaccines, gene-loaded systems, and reformulated chemotherapies. These interventions, underpinned by molecular design principles, have demonstrated superior tumor targeting, enhanced tolerability, and improved response rates compared to conventional modalities. Notably, this era marked the first regulatory approvals of NP therapies in lung cancer, accompanied by early successes in improving patient outcomes, underscoring a tangible impact from the laboratory to the clinic. Further clinical trial details are summarized in Table 2.

**Table 2:** Summary of clinical trials (2020–2025) in lung cancer nanomedicine.

**Nanomedicine platform**	**Clinical stage/population**	**Findings**	**Reference(s)**
Telisotuzumab vedotin (Teliso-V)—c-MET-targeted ADC	Phase II (LUMINOSITY) in advanced NSCLC with high c-MET overexpression (*EGFR* wild-type); FDA ACC 2023.	ORR 35% (all PR) in c-MET IHC 3+ patients; median DOR 7.2 months. Responses enriched in c-MET high (e.g., ~54% ORR in IHC 3+ subgroup). Common AEs: nausea, fatigue, hematologic toxicity (mostly Grade 1–2). First c-MET-directed ADC approved in lung cancer, addressing an unmet need in c-MET-overexpressed NSCLC.	[88]
Datopotamab deruxtecan (Dato-DXd)—*TROP2*-targeted ADC	Phase III (TROPION-Lung01) in metastatic NSCLC post-1L (*TROP2*-expressing); results 2023–2024.	Met primary PFS endpoint (significant improvement vs. docetaxel). In non-squamous NSCLC: OS 14.6 vs. 12.3 mo (HR 0.84, trend); higher ORR and longer DOR than chemo (data pending publication). Grade ≥ 3 TRAEs 26% vs. 42% (less toxicity vs docetaxel). First *TROP2* ADC to show survival benefit trend in NSCLC.	[91]
*HER3*-DXd—*HER3*-targeted ADC	Phase II (HERTHENA-Lung01) in *EGFR*-mutant NSCLC after *EGFR* TKI ± chemo; phase III trial ongoing.	Phase II: ORR ~30% in heavily pretreated *EGFR* TKI-resistant patients (including ~33% ORR in those with brain metastases); median PFS ~5.5 mo, OS ~11.9 mo. Early phase III data (HERTHENA-Lung02) vs. chemo show improved PFS (5.8 vs. 5.4 mo, HR 0.77) and ORR (35% vs. 25%) but no OS difference (16.0 vs. 15.9 mo). High rate of Grade ≥ 3 AEs (~65%) observed with monotherapy. Demonstrated intracranial activity and efficacy across diverse resistance mechanisms; further combination strategies under investigation.	[76, 92]
T-DXd—HER2-targeted ADC	Phase II (DESTINY-Lung01) in advanced HER2-mutant NSCLC after chemo; FDA-approved under ACC.	ORR ~55%, median PFS ~8.2 mo, OS ~17.8 mo. Notable toxicity: ILD in 26% (Grade ≥ 3 in ~10%, 2 % fatal). First HER2-directed therapy in lung cancer, showing high efficacy in HER2-mutant NSCLC.	[93]
Disitamab vedotin (RC48)	Advanced HER2-mutant NSCLC (phase II, China).	Demonstrated encouraging efficacy in patients with HER2-mutant or overexpressing NSCLC, showing an ORR of 45.5% and a median PFS of 7.5 mo. In subgroups treated with platinum-based chemo ± bevacizumab, ORR improved to 71.4%. Treatment was generally well tolerated with mostly mild-to-moderate adverse events.	[94]
Rovalpituzumab tesirine (Rova-T)	Phase III in DLL3-high SCLC (TAHOE trial).	Failed to improve OS vs. topotecan (6.3 vs. 8.6 mo). Associated with significant toxicity, including pleural and pericardial effusions, leading to early termination of the trial due to futility.	[95]
*EGFR*-targeted bacterial nanocell (EDV^TM^)—biomimetic	Phase I/IIa (2025, *EGFR*-positive refractory solid tumors, including NSCLC; ≥ 2L basket trial).	Earlier phase I studies showed acceptable safety for EDV nanocells in advanced cancers. A new multicenter trial is underway delivering *EGFR*-targeted EDV^TM^ loaded with a “super-cytotoxic” drug (PNU-159682) plus an immunostimulant (α-GalCer) to *EGFR*-expressing tumors. Will evaluate safety/efficacy in patients who have exhausted standard 2L therapies (including NSCLC). (No efficacy results yet; prior EDV trials in other cancers reported minimal side effects and signs of overcoming drug resistance.)	[95, 96]
mRNA-lipid nanoparticle (mRNA-LNP) vaccine—BNT116	Phase I (LuCa-MERIT-1) in advanced NSCLC; fixed six-antigen mRNA vaccine, as monotherapy or with chemo/immunotherapy.	Immunogenicity demonstrated: vaccine-induced CD8^+^ and CD4^+^ T-cell responses to multiple tumor antigens in patients. In combination (with docetaxel or anti-PD-1), early signs of efficacy were observed (including some partial responses in heavily pretreated patients). Safety profile was acceptable: mostly low-grade cytokine release symptoms and injection-site reactions; no dose-limiting toxicities. Represents the first-in-human mRNA cancer vaccine in NSCLC, showing feasibility and preliminary anti-tumor activity.	[97, 98]
NBTXR3 hafnium oxide nanoparticle—radioenhancer (intratumoral)	Phase I (MD Anderson) in inoperable, locally recurrent NSCLC (with re-irradiation ± anti-PD-1).	Feasible CT-guided intratumoral injections; established recommended phase II dose with a favorable safety profile (no dose-limiting toxicities). The ongoing expansion phase is evaluating efficacy; preliminary signals indicate local tumor control and potential immune activation (abscopal effects reported in other NBTXR3 studies). First-in-class inorganic nanotherapeutic activating radiotherapy, aiming to improve outcomes in radioresistant lung tumors.	[99]
Hafnium-oxide nanoparticles (NBTXR3)—stimuli-responsive radioenhancer	Phase I (ongoing; stage III NSCLC unsuitable for surgery, intratumoral NBTXR3 + radiotherapy ± anti-PD-1).	A novel inorganic nanoparticle that amplifies radiotherapy effects. Preliminary findings (2025 ELCC) show intratumoral NBTXR3 (activated by radiation) can enhance local tumor control without increasing systemic toxicity. Early safety data in thoracic tumors are encouraging (few ≥ Grade 3 AEs, mainly procedure-related, e.g., one pneumothorax) and demonstrate the feasibility of intratumoral injections in lung lesions. Moreover, abscopal responses have been documented in trials—untreated lesions regressing post-therapy—suggesting NBTXR3 plus hypofractionated radiation may stimulate systemic anti-tumor immunity.	[100]
Nab-paclitaxel (albumin-bound) vs. docetaxel	Advanced NSCLC, 2L vs. docetaxel (Phase III, 503 pts).	Median OS ~16 months in the nab-paclitaxel arm, non-inferior to docetaxel. Notably, severe neutropenia was much lower with nab-paclitaxel (febrile neutropenia 2% vs. 22% on docetaxel), indicating a significantly improved safety profile at similar efficacy.	[11]
NALIRI (vs. topotecan)	Phase III (RESILIENT) in 2L SCLC.	ORR was significantly higher with NALIRI (44.1% vs. 21.6%), but no significant improvement in OS or PFS. Hematologic toxicities were less frequent in the liposomal arm, supporting a more favorable safety profile.	[101]
Pm-Pac—cremophor-free micellar taxane	Phase III (open-label) in untreated advanced NSCLC (Pm-Pac + cisplatin vs. solvent paclitaxel + cisplatin).	Pm-Pac arm achieved higher ORR (by independent review) and longer median PFS (6.4 vs. 5.3 mo, HR 0.63, *p* = 0.0001). No OS difference. Safety improved: serious AEs 9% vs. 18%; significantly less Grade ≥ 3 neutropenia (1% vs. 23%). Validated nanoparticle delivery (no CrEL solvent) improves efficacy and tolerability in first-line NSCLC.	[73]
Inhalable lipid-polymer hybrids; sapanisertib + telaglenastat	Phase I in advanced NSCLC; phase I in NSCLC with KEAP1/NFE2L2 mutations.	Safe lung-specific delivery, minimal systemic exposure; ORR 12.5%; 1 PR in squamous NRF2-mutant patient. Well tolerated.	[44]
NALIRI—PEGylated liposome SN-38	Phase II (dose exploration) in relapsed small-cell lung cancer (2L after platinum)—China.	ORR 32% (21/66 pts; 95% CI 21–44) across doses; median PFS 4.0 mo, OS 9.7 mo. Recommended dose 80 mg/m^2^ had the best risk-benefit (ORR 37%, manageable diarrhea). Grade ≥ 3 TRAEs in 47% (mostly neutropenia 27%, leukopenia 24%, anemia 15%). Demonstrated activity of nanoparticle irinotecan in SCLC, though phase III (RESILIENT) showed no OS benefit vs. topotecan.	[72]
Oncoprex (REQORSA)—TUSC2 gene-loaded liposome	Phase I/II in advanced NSCLC (monotherapy or combined with *EGFR*-TKI/ICI)	First-in-human systemic delivery of tumor suppressor gene via nanoparticle. Phase I showed safety and early efficacy (PR/stable disease). Ongoing phase II evaluating combinations.	[102]
CRISPR-edited PD-1 knockout T cells	Phase I in NSCLC (China)	Established the feasibility of CRISPR-edited T cell therapy in solid tumors. No cytokine release syndrome observed. Edited cells persisted in circulation and showed early signs of immune activity.	[103]

MET: mesenchymal-epithelial transition; ADC: antibody-drug conjugate; NSCLC: non-small cell lung cancer; *EGFR*: epidermal growth factor receptor; ACC: accelerated approval; ORR: objective response rate; PR: partial response; IHC: immunohistochemistry; DOR: duration of response; AEs: adverse events; *TROP2*: trophoblast cell surface protein 2; PFS: progression-free survival; OS: overall survival; HR: hazard ratio; mo: month; chemo: chemotherapy; TRAEs: treatment-related adverse events; *HER3*: human epidermal growth factor receptor 3; TKI: tyrosine kinase inhibitor; ILD: interstitial lung disease; DLL3: delta-like ligand 3; SCLC: small cell lung cancer; 2L: second-line; Pm-Pac: polymeric micellar paclitaxel; CrEL: cremophor el solvent; PEG: polyethylene glycol; ICI: immune checkpoint inhibitor.

## Limitations and future perspectives

Despite the impressive advances in lung cancer nanomedicine, significant limitations remain that hinder widespread clinical translation. NP instability remains a core issue, as many formulations aggregate or degrade during storage or circulation, altering their physicochemical properties and reducing efficacy. This challenge is particularly pronounced for biologic carriers such as exosomes, which require ultracold preservation to remain viable [104]. Similarly, magnetic NPs used in theranostics often face stability issues due to strong dipole-dipole interactions that drive aggregation. A recent strategy to overcome this is the magnetite nanoring morphology, which adopts a vortex magnetization that quenches interparticle interactions. Importantly, these nanorings maintain hyperthermia efficiency while enabling controlled heating within the 42–45°C apoptotic window, offering a promising route to safer and more effective magnetic nanocarriers [105].

Biodistribution variability is another key limitation. The EPR effect is highly inconsistent in human lung tumors, and systemically delivered NPs are often cleared by the mononuclear phagocyte system or sequestered in non-target tissues. As a result, therapeutic accumulation at the tumor site may be insufficient, with an increased risk of off-target toxicity. Immunogenicity and long-term toxicity also remain concerns, particularly for inorganic or carbon-based materials [106].

In addition, although nanomedicine is generally considered well-tolerated, several clinical studies in lung cancer have reported rare but serious adverse events. For instance, T-DXd was associated with an adjudicated drug-related interstitial lung disease (ILD) incidence of 26%, including fatal cases, in a phase II trial of HER2-mutant NSCLC, necessitating steroid intervention and treatment discontinuation in some patients [93]. Similarly, the DLL3-targeted ADC Rova-T for small-cell lung cancer exhibited unique toxicities, including pleural and pericardial effusions and fatal events such as pneumonitis and organ damage, ultimately leading to early termination of its clinical development [95]. These observations indicate that while life-threatening side effects of nanotherapies in lung cancer remain uncommon, they are clinically significant and warrant vigilant monitoring and risk mitigation in future trials.

Conventional animal models often fail to recapitulate human immunity and the TME. In parallel, the regulatory framework for nanomedicine is still underdeveloped. There is no universal definition or set of quality standards for nanoformulations, and approval is often handled on a case-by-case basis. Regulatory agencies, such as the FDA and European Medicines Agency (EMA), have recently begun issuing draft guidance; however, the lack of harmonized standards continues to pose barriers. Manufacturing is also a challenge, as scale-up from laboratory synthesis to Good Manufacturing Practice (GMP) production can alter NP properties and reduce reproducibility. Technologies such as microfluidic NP synthesis are now being utilized to enhance batch-to-batch consistency [104]; however, broader adoption is still needed.

Despite these barriers, several strategies show promise. Biologically derived nanocarriers such as exosomes can be engineered for targeted delivery with high biocompatibility [107]. Inhalable nano-immunotherapies, including aerosolized nanovaccines and checkpoint inhibitors, are being developed to trigger lung-specific immune responses while minimizing systemic toxicity [108]. Artificial intelligence (AI) is increasingly used to optimize nanocarrier design, as machine learning (ML) algorithms now help predict how structural features affect biodistribution and clearance, enabling the rational tailoring of NPs for the lung [109]. AI/ML frameworks are expected to improve predictive modeling of in vivo behavior. In particular, NP design and the prediction of nanomedicine biodistribution are expected to be among the first areas to benefit from AI/ML by 2030, underscoring their prospective impact on the field [110]. Furthermore, personalized nanomedicine is on the horizon. Patient-specific nanovaccines have demonstrated potent, polyclonal T-cell responses and durable memory in lung tumor models [63], while NP-mediated CRISPR-Cas9 delivery has successfully achieved gene editing in the lung without the use of viral vectors. These advances may open new frontiers in treating resistant or genetically complex lung cancers. On a translational level, real-world data are accumulating. For example, nab-paclitaxel is now approved for first-line treatment for advanced NSCLC, with improved tolerability and efficacy over solvent-based formulations [111]. At the same time, setbacks like BIND-014 highlight the need for robust trial design and biomarker-guided patient selection [112]. Success will require interdisciplinary collaboration between scientists, clinicians, and regulators, as well as early engagement with agencies to preemptively address concerns such as immunogenicity and cost-effective manufacturing. If these hurdles are actively addressed, lung cancer nanomedicine is well-positioned to evolve from experimental innovation into a clinical standard.

## Conclusions

Lung cancer therapeutics are shifting with the integration of molecularly engineered nanotechnology. A growing repertoire of platforms, including inhalable nanocarriers, stimuli-responsive theranostics, and bio-inspired immunomodulatory systems, exemplifies how precise molecular architecture and functionality are being harnessed to overcome long-standing barriers in drug delivery. These nanocarriers are not only designed to achieve tumor specificity but also to respond dynamically to microenvironmental cues, enabling the spatiotemporal control of release and synergistic therapeutic effects. This review highlights how rational molecular design translates into improved pharmacokinetics, tumor penetration, and immunomodulation. In particular, inhalable nanoformulations illustrate how molecularly optimized delivery routes can achieve localized efficacy with reduced systemic burden, enhancing both safety and patient compliance. As several of these nanomedicine strategies progress toward clinical translation, sustained efforts in biomolecular characterization, scalable synthesis, and regulatory standardization will be essential. Moreover, interdisciplinary collaborations bridging materials science, molecular oncology, and clinical research will be critical to fully realize the therapeutic promise of these platforms. Ultimately, molecular-level innovation in nanomedicine is poised to transform lung cancer therapy toward more precise, personalized, and less toxic interventions.

## Abbreviations

ADCs: antibody-drug conjugates
AI: artificial intelligence
*ALK*: anaplastic lymphoma kinase
*CDK4*: cyclin-dependent kinase 4
CRISPR: clustered regularly interspaced short palindromic repeats
CRISPR-Cas9: clustered regularly interspaced short palindromic repeats-associated protein 9
Dato-DXd: datopotamab deruxtecan
DLL3: delta-like ligand 3
DMSA: dimercaptosuccinic acid
*EGFR*: epidermal growth factor receptor
EPR: enhanced permeability and retention
FDA: Food and Drug Administration
GSH: glutathione
*HER3*: human epidermal growth factor receptor 3
ICD: immunogenic cell death
MDR: multidrug resistance
ML: machine learning
MMP-2: matrix metalloproteinase-2
MRI: magnetic resonance imaging
NALIRI: liposomal irinotecan
NIR: near-infrared
NPs: nanoparticles
NSCLC: non-small-cell lung cancer
PD-1: programmed cell death protein 1
PD-L1: programmed death-ligand 1
PLGA: poly lactic-co-glycolic acid
*PLK1*: polo-like kinase 1
Pm-Pac: polymeric micellar paclitaxel
ROS: reactive oxygen species
Rova-T: rovalpituzumab tesirine
siRNA: small interfering RNA
SPIONs: superparamagnetic iron oxide nanoparticles
STING: stimulator of interferon genes
TAMs: tumor-associated macrophages
TME: tumor microenvironment
*TROP2*: trophoblast cell surface protein 2

## Declarations

### Copyright

© The Author(s) 2025.
